# Maximum Lifetime Body Mass Index and Mortality in Mexican American Adults: the National Health and Nutrition Examination Survey III (1988–1994) and NHANES 1999–2010

**DOI:** 10.5888/pcd14.160588

**Published:** 2017-08-17

**Authors:** Carrie R. Howell, Kevin Fontaine, Keisuke Ejima, Kirsten K. Ness, Andrea Cherrington, Tapan Mehta

**Affiliations:** 1 Department of Epidemiology and Cancer Control, St. Jude Children's Research Hospital, Memphis, Tennessee; 2 Department of Health Behavior, School of Public Health, The University of Alabama at Birmingham, Birmingham, Alabama; 3 Office of Energetics, School of Health Professions, The University of Alabama at Birmingham, Birmingham, Alabama; 4 Institute of Industrial Science, University of Tokyo, Tokyo, Japan; 5 Division of Preventive Medicine, School of Medicine, The University of Alabama at Birmingham, Birmingham, Alabama; 6 Department of Health Services Administration, School of Health Professions, The University of Alabama at Birmingham, Birmingham, Alabama

## Abstract

**Introduction:**

Studies in US Hispanic adults indicate no deleterious association between obesity and death. We tested the hypothesis that accounting for weight history would provide more insight into this nonassociation.

**Methods:**

We used the National Health and Nutrition Examination Survey (NHANES) to examine associations between maximum lifetime body mass index (BMI) and all-cause and cause-specific mortality among US-residing Mexican American adults. BMI was classified as underweight (<18.5 kg/m^2^), normal weight (18.5–24.9), overweight (25.0–29.9), obese class I (30.0–34.9), and obese class II (≥35.0). We used Cox proportional hazards to examine the association between maximum lifetime BMI and BMI at survey and all-cause and specific causes of death (ie, cardiovascular disease, cancer, diabetes, and other) controlling for age, sex, and smoking in 6,242 Mexican American adults enrolled in NHANES III (1988–1994) and NHANES 1999–2010.

**Results:**

Mexican Americans categorized as obese class II at maximum lifetime and time of survey had increased risk of all-cause mortality (hazard ratio [HR], 2.12; 95% confidence interval [CI], 1.54 - 2.93 and HR, 1.52; 95% CI, 1.10–2.10). Those reporting a maximum lifetime BMI of class I or class II obesity but who were classified as normal weight at survey had increased risk of all-cause mortality (HR = 2.49; 95% CI, 1.72–3.61 and HR = 3.56; 95% CI, 1.15–11.06, respectively).

**Conclusion:**

Increased all-cause mortality risk in Mexican Americans with a lifetime BMI of 35 or greater refutes prior studies, suggesting that maximum lifetime BMI should be included when evaluating obesity–mortality associations in this population.

## Introduction

The US-residing Hispanic population has increased by 43% in the last decade (1), and age-adjusted data from 2011 through 2012 indicate that 77.9% of Hispanic adults were overweight or obese, compared with 67.2% of their white, non-Hispanic counterparts (2). Studies have shown that body mass index (BMI) is associated with death in white and black populations and that those classified as underweight or obese have higher rates of all-cause mortality than those at a normal weight (3,4). However, this association has not been observed in US-residing Hispanic adults (5,6).

We re-examined this lack of an association for several reasons. First, previous studies used older mortality data (5) and self-reported BMI (6). Since conducting these analyses, the National Center for Health Statistics (NCHS) released updated mortality data (through December 31, 2011) for the National Health and Nutrition Examination Survey (NHANES) and additional years of continuous NHANES data including measured BMI. Second, a significant limitation in BMI–death analyses is the issue of reverse causality (7–9), where estimates may be confounded by individuals who have lost weight because of underlying illness or intentional weight loss (10). Recently, investigators have proposed that using an individual’s maximum lifetime weight before the time of data collection accounts for reverse causality and produces estimates that more accurately capture the obesity–death association (8,9). Third, previous BMI–death analyses among Hispanics have concentrated on death from all causes (5,6). Focusing on all-cause mortality may mask significant associations with specific causes of death. For example, obese Hispanics have higher rates of death from diabetes (11) and cardiovascular disease (CVD) (12) than nonobese Hispanics. Given these possibilities, the aim of this study was to examine the association between maximum lifetime BMI and all-cause and cause-specific mortality among US-residing Mexican American adults in NHANES III (1988–1994) and the continuous NHANES 1999–2010. We hypothesized that obesity was associated with an increased risk of death in US-residing Mexican American adults.

## Methods

### Data sources and inclusion criteria

Data for these analyses were drawn from 2 nationally representative surveys: NHANES III (1988–1994) and the continuous NHANES 1999–2010. Baseline data from these surveys were linked to mortality follow-up data (through December 31, 2011) for analyses. NHANES examines the health status of a nationally representative sample of US residents (13), with data collected via both self-report questionnaires and physical examinations. Public-use mortality data for the continuous NHANES are probabilistically matched to the National Death Index records through 2011 and are publicly available from the Centers for Disease Control and Prevention (CDC) (13). This project was declared exempt by the University of Alabama at Birmingham Institutional Review Board.

Mexican American adults were included in the analyses if they participated in either NHANES III or NHANES 1999–2010, were aged 20 years or older at the time of participation, had measured height and weight variables to calculate BMI, answered a question based on maximum lifetime BMI, and had cause-specific mortality information (Figure). Because the sampling of non-Mexican Hispanic American participants in NHANES III and NHANES 1999–2007 was proportionately smaller than the US population, NCHS (14) recommends limiting analyses to Mexican Americans for NHANES before 2007 to produce the most precise estimates. For this reason, we limited our analyses to Mexican American participants.

**Figure:**
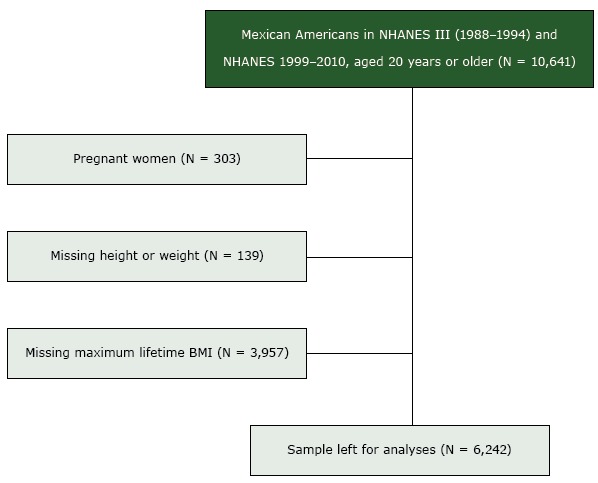
Consolidated Standards of Reporting (CONSORT) diagram describing the flow of participants selected for inclusion in the study of maximum lifetime body mass index (BMI) and all-cause and cause-specific mortality in Mexican American adults: National Health and Nutrition Examination Survey (NHANES) III, (1988–1994) and 1999–2010. Pregnant women and those missing height, weight, or maximum lifetime body mass index were excluded.

### Study variables

Maximum lifetime BMI was constructed by using self-reported maximum lifetime weight (based on an NHANES question that asks participants to recall maximum weight) and measured height at baseline. BMI at survey was calculated from measured height and weight (collected during the physical examination portion of NHANES) at the time of survey. Both BMI variables were categorized according to the National Heart, Lung, and Blood Institute’s BMI classifications as used in previous analyses (5,6). The classifications were underweight (<18.5 kg/m^2^), normal weight (18.5–24.9 kg/m^2^), overweight (25.0–29.9 kg/m^2^), obese class I (30.0–34.9 kg/m^2^), and obese class II (≥35.0 kg/m^2^).

Age at all-cause death and age at cause-specific death or censor date (December 31, 2011) were used as outcome variables, taking into account competing risks by coding deaths from other causes as censored at time of death for each respective cause-specific death. Cause-specific deaths were categorized on the basis of the top 10 leading causes of death created by NCHS based on comparable groups from the *International Statistical Classification of Diseases and Related Health Problems*, 10th revision (ICD–10). Because of the low number of events in the Mexican American sample, deaths were collapsed into 4 cause-specific categories: deaths from CVD (ICD–10 codes I00–I09, I11, I13, I20–I51), cancer deaths (ICD–10 codes C00-C97), diabetes deaths (ICD–10 codes E10–E14), and all other causes of death (residual). A review of the death tables for Hispanics found that the cause-specific death information collected in NHANES covered the top 10 leading causes of death for Hispanics in 2010 (15) with the collapsed categories representing 3 of the top 5 leading causes of death (from cancer, from heart disease, and diabetes-related). The other top 10 leading causes of death were categorized as “all other causes” and included chronic lower respiratory diseases; accidents; Alzheimer’s disease; influenza and pneumonia; nephritis, nephrotic syndrome, and nephrosis; and other deaths.

Covariates were baseline age, sex, and smoking status. Age at baseline was treated as a continuous variable in the model to adjust for years before study entry (16). Smoking status was categorized as current, former, or never for all data and treated categorically in the models.

### Statistical analysis

Hazard ratios were calculated using Cox proportional hazard regression with age at death or censoring as the time scale (16). Hazard ratios and 95% confidence intervals were estimated for all-cause mortality and each cause-specific death risk for each stable BMI category and weight loss category where an individual reported a higher maximum lifetime BMI and a lower BMI at the time NHANES was conducted. Normal weight adults who remained stable were used as the reference group for estimates. We also estimated hazard ratios and 95% confidence intervals (CIs) for all-cause and each cause-specific death risk for each BMI at survey category for comparison purposes. Appropriate weights were calculated and applied as instructed by CDC (17) in each analytical model.

Separate models were fitted for all-cause mortality and each cause-specific death, taking into account competing risks by coding deaths from other causes as censored at time of death for each respective model (18) and included the covariates of baseline age, sex, and smoking status. Proportional hazards assumptions where checked using weighted Schoenfeld residuals (19) correlated with the log of the time scale (age at death or censor). For covariates that appeared to not satisfy the proportional hazards assumptions via Schoenfeld residuals, weighted Kaplan–Meier curves were plotted and examined and indicated that the assumptions were adequately satisfied. Missing data were handled by using listwise deletion. R version 3.3.1 (Microsoft Corporation) and its library survey was used to conduct all analyses.

## Results

We identified 10,641 unweighted potentially eligible Mexican Americans in NHANES III and NHANES 1999–2010. After excluding data from some respondents, we had an unweighted sample of 6,242 Mexican American adults for analysis. The mean baseline age in years was 43.1 (standard deviation [SD], 16.4 y) and the mean mortality follow-up in years was 15.5 (SD, 6.7 y). The mean maximum lifetime BMI was 29.8 (SD, 6.0) kg/m^2^ and the mean BMI at survey was 28.0 (SD, 5.5) kg/m^2^. Approximately 48% of the sample was female, 55% were never smokers, and there were 1,196 unweighted deaths overall, of which 94 were CVD-related, 255 were cancer-related, 91 were diabetes-related, and 756 were due to all other causes.

The categorization of individuals in the data set by BMI at survey and maximum lifetime BMI are described in Table 1. Approximately 70% of the Mexican Americans in this sample remained in their respective BMI weight classes at maximum lifetime BMI and at time of survey, and the remaining almost 30% had a change in BMI weight class where maximum lifetime BMI was higher than at time of survey.

**Table 1:** Maximum Lifetime Body Mass Index (BMI) and BMI at Survey Among Mexican American Adults, National Health and Nutrition Examination Survey III (1988–1994) and NHANES 1999–2010

BMI at Survey^a^	Maximum Lifetime BMI^a^, No.					
	Underweight	Normal Weight	Overweight	Obese Class I	Obese Class II	Total
Underweight	8	42	7	1	0	58
Normal weight	—	1,021	619	94	22	1,756
Overweight	—	—	1,710	657	95	2,462
Obese class I	—	—	—	1,025	300	1,325
Obese class II	—	—	—	—	641	641
**Total**	8	1,063	2,336	1,777	1,058	6,242

Abbreviation: —, not applicable.

a Underweight (<18.5 kg/m^2^), normal weight (18.5–24.9 kg/m^2^), overweight (25.0–29.9 kg/m^2^), obese class I (30.0–34.9 kg/m^2^), obese class II (≥35.0 kg/m^2^).

### All-cause mortality

By using BMI at survey only we found no significant increased risk of death with excess weight compared with normal weight Mexican Americans (Table 2). Mexican Americans who reported a maximum lifetime BMI of class I obese or class II obese but were classified as normal weight at time of survey had an increased risk of all-cause mortality compared with those with a maximum lifetime BMI of normal weight who were classified as normal weight at the time of the survey (HR, 2.49; 95% confidence interval [CI], 1.72–3.61 and HR, 3.56; 95% CI, 1.15–11.06, respectively) (Table 3). Furthermore, individuals who reported a maximum lifetime BMI of class II obese but were classified as overweight at time of survey had an increased risk of death (HR, 2.76, 95% CI, 1.74–4.37) compared with those with a maximum lifetime BMI of normal weight who were classified as normal weight at the time of the survey. Those who lost weight and moved from class II obese to class I obese over time had a significant increased risk of all-cause death (HR, 2.12; 95% CI, 1.54–2.93) compared with those with a maximum lifetime BMI of normal weight who were classified as normal weight at the time of the survey. Mexican Americans who remained stable in the obese class II category had an increased risk of death (HR, 1.52; 95% CI, 1.10–2.10). However, individuals who remained stable in the overweight, and obese class I categories from maximum lifetime BMI to BMI at time of survey did not have a significant increased risk of death compared with those with a maximum lifetime BMI of normal weight and were classified as normal weight at the time of the survey.

**Table 2:** Hazard Ratios and 95% Confidence Intervals^a^ for All-Cause and Cause-Specific Mortality Among Mexican American Adults Based on Body Mass Index (BMI) at Survey, National Health and Nutrition Examination Survey III (1988–1994) and NHANES 1999–2010

Survey BMI^b^	All Causes^c^	Cardiovascular Disease	Cancer	Diabetes	All Other Causes^d^
	Hazard Ratio (95% Confidence Interval)				
Normal weight	1 [Reference]				
Overweight	0.97 (0.75–1.24)	0.97 (0.43–2.19)	0.97 (0.64–1.48)	1.10 (0.65–1.86)	0.95 (0.74–1.24)
Class I obese	1.19 (0.91–1.56)	1.23 (0.55–2.77)	0.89 (0.52–1.50)	1.75 (0.91–3.36)	1.28 (0.87–1.88)
Class II obese	1.25 (0.90–1.72)	1.01 (0.34–2.97)	0.84 (0.52–1.36)	2.09 (0.93–4.69)	1.39 (0.99–1.96)

a Adjusted for age, sex, and smoking status.

b Underweight (<18.5 kg/m^2^), normal weight (18.5–24.9 kg/m^2^), overweight (25.0–29.9 kg/m^2^), obese class I (30.0–34.9 kg/m^2^), obese class II (≥35.0 kg/m^2^).

c Deaths from any cause.

d Deaths from chronic lower respiratory diseases; accidents; Alzheimer’s disease; influenza and pneumonia; nephritis, nephrotic syndrome, and nephrosis; and any other deaths other than those related to cardiovascular disease, cancer, or diabetes.

**Table 3:** Hazard Ratios^a^ and 95% Confidence Intervals^a^ for All-Cause and Cause-Specific Mortality Among Mexican American Adults Based on Maximum Lifetime Body Mass Index (BMI), National Health and Nutrition Examination Survey, 1999–2010

Maximum Lifetime BMI^b^	Survey BMI^b^	All-Causes^c^	Cardiovascular Disease	Cancer	Diabetes	All Other Causes^d^
		Hazard Ratio (95% Confidence Interval)				
Normal weight	Normal	1 [Reference]				
Overweight	Normal	1.21 (0.88–1.66)	0.50 (0.18–1.35)	1.29 (0.51–3.23)	0.94 (0.28–3.09)	1.34 (0.93–1.92)
Class I obese	Normal	2.49 (1.72–3.61)	0.75 (0.16–3.61)	2.4 (0.64–9.03)	5.86 (1.16–29.70)	2.63 (1.57–4.39)
Class II obese	Normal	3.56 (1.15–11.06)	1.71 (0.16–18.48)	1.08 (0.15–7.66)	3.23 (0.26–40.02)	5.2 (1.52–17.78)
Overweight	Overweight	1.08 (0.81–1.43)	0.80 (0.26–2.46)	1.06 (0.55–2.04)	0.76 (0.22–2.59)	1.16 (0.83–1.63)
Class I obese	Overweight	1.25 (0.92–1.70)	0.68 (0.28–1.68)	1.48 (0.83–2.62)	1.79 (0.57–5.61)	1.21 (0.85–1.73)
Class II obese	Overweight	2.76 (1.74–4.37)	0.22 (0.03–1.80)	0.59 (0.16–2.14)	13.42 (4.27–42.20)	3.37 (1.85–6.14)
Class I obese	Class I Obese	1.23 (0.88–1.71)	0.43 (0.18–1.06)	0.91 (0.49–1.69)	1.28 (0.42–3.89)	1.52 (0.97–2.40)
Class II obese	Class I Obese	2.12 (1.54–2.93)	2.47 (0.78–7.81)	1.55 (0.83–2.90)	5.17 (1.34–19.96)	2.05 (1.29–3.26)
Class II obese	Class II Obese	1.52 (1.10–2.10)	0.78 (0.25–2.43)	1.02 (0.51–2.02)	2.73 (0.65–11.48)	1.81 (1.29–2.54)

a Adjusted for age, sex, and smoking status.

b Underweight (<18.5 kg/m^2^), normal weight (18.5–24.9 kg/m^2^), overweight (25.0–29.9 kg/m^2^), obese class I (30.0–34.9 kg/m^2^), obese class II (≥35.0 kg/m^2^).

c Deaths from any cause.

d Deaths from chronic lower respiratory diseases; accidents; Alzheimer’s disease; influenza and pneumonia; nephritis, nephrotic syndrome, and nephrosis; and any other deaths other than those related to cardiovascular disease, cancer, or diabetes .

### Cause-specific mortality

By using BMI at survey only, we found no significant increased risk of any cause-specific death among Mexican Americans with excess weight compared with those of normal weight (Table 2). Mexican Americans who reported a maximum lifetime BMI of class I obese but were classified as normal weight at time of survey and those who reported a maximum lifetime BMI of class II obese but were classified as overweight at time of survey had an increased risk of diabetes-related death (HR, 5.86; 95% CI, 1.16–29.70 and HR, 13.42; 95% CI, 4.27–42.20, respectively) (Table 3). Those who lost weight and moved from class II obese to class I obese over time also had a significant increased risk of diabetes-related death (HR, 5.17; 95% CI, 1.34–19.96). Similar findings were found for deaths from all other causes. We found no significant increased risk of CVD-related and cancer-related deaths in this cohort.

## Discussion

Overall, we found that Mexican Americans whose weight remained stable did not have an increased risk of all-cause or cause-specific mortality due to excess weight. Likewise, when using BMI at survey only in the models, we did not observe an increased risk of all-cause or cause-specific mortality due to excess weight. However, we did find that Mexican Americans who reported a maximum lifetime BMI of class II obese had a slightly increased risk of death than those who maintained a normal weight over time. Interestingly, we found the associations of the highest magnitude between excess weight and mortality in Mexican Americans who lost weight between the point in time where they experienced their maximum weight to the time of survey, particularly among those who were class II obese. Estimates for diabetes-related and all other causes of death outside of CVD and cancer produced similar trends among those who lost weight over time.

For the estimates for Mexican Americans that remained in a stable weight category, our findings are similar to those of Fontaine et al (5) and Mehta et al (6) where there was no association between increased risk of death and the overweight or class I obese category in Hispanic populations. Furthermore, our estimates using the approach of BMI at survey only also confirmed previous findings. However, our finding that Mexican Americans who remained stable at a BMI of 35 kg/m^2^ or greater had a significant increased risk of all-cause mortality is in contrast to previous results. Our analysis explained previous findings of reverse causality by teasing out those who lost weight over time (whether unintentionally or intentionally), producing estimates that capture the association between BMI and death given that an individual remained stable in the class II category over time. Considering that this sample contained almost 30% of individuals who moved from a higher BMI category to a lower BMI category over time, similar to a rate reported by Stokes and Preston (9) in another NHANES sample, previous estimates have likely been confounded by this group of individuals who lost weight.

Our finding that Mexican Americans who lost weight over time had a significant increased risk of all-cause and diabetes-related mortality mirror those of Stokes and Preston (9) who found similar results in an older sample of adults in NHANES. They found that individuals who reported a maximum lifetime BMI as class II obese or class I obese had an increased risk of death (HR = 3.18, 95% CI, 2.09–4.83 and HR = 1.81, 95% CI: 1.16–2.82, respectively). Our results appear to confirm that individuals losing weight over time, possibly because of illness or through intent, are biasing the estimates in previous analyses. For instance, after accounting for individuals who lost weight, we find a significant association between individuals who were class II obese and death in those who remained stable, again similar to Stokes (8) who found a strengthening association for those who were class II obese once he accounted for those who lost weight in the sample.

A similar trend of higher risk for those who lost weight and who had a diabetes-related death support the contention that lifetime BMI is likely a better predictor of death than the most recent measure. One reason for this finding could be that patients with poorly controlled diabetes can lose weight (unintentionally) as the body breaks down muscle and fat for energy since it cannot process sugar for energy. Furthermore, individuals diagnosed with diabetes have likely been counseled to lose weight; however, diabetes is a chronic illness and the risk of death is likely higher because of diabetes-related illness. We also found that those who lost weight and moved from class II obese to class I obese over time had an increased risk of diabetes-related death. This is similar to findings of Rogers et al (20) who found that among US adults in the National Health Interview Survey who had a diabetes-related death, those who were class I obese had a 2.8-fold increased risk of death and those who were class II obese had a 4-fold increased risk compared with normal weight individuals. Moreover, Hispanics may be at higher risk of death related to diabetes regardless of BMI. For instance, Kposowa (21) used data from the US National Longitudinal Mortality Study and found that Hispanics had a 28% increased risk of diabetes-related deaths compared with non-Hispanic whites.

Additionally, we found no increased risk of cancer or CVD-related deaths by using the BMI at survey or the maximum lifetime BMI approach in this population. Although CVD has been associated with an increased risk of death (3), estimates specific to Mexican Americans have not been previously reported, and the obesity–CVD mortality association may be different in this ethnic population. In terms of cancer-related deaths, our results are similar to those of a recent study that showed a lack of an association between increased weight and breast cancer–related deaths in Hispanic women (22).

These analyses have several strengths. First, it is the first analysis to use a self-reported maximum lifetime BMI approach to modeling the association between excess weight and death in a group of Mexican American adults. Second, our approach accounted for a major limitation of potential reverse causality in previous analyses of this nature where we were able to classify and produce separate estimates for those individuals that may be at a lower BMI at the time of survey (7) because of advanced disease states or intentional weight loss. Third, we were able to use a large nationally representative sample of Mexican Americans with nearly 16 years of mortality follow-up data, producing the most precise estimates to date. Fourth, we used cause-specific mortality as an outcome which, to our knowledge, has not yet been examined specifically with regards to the association between obesity and mortality in Mexican American adults.

Our study also has several limitations. First, maximum lifetime weight was self-reported and subject to recall bias, which can influence BMI-related estimates (23). Second, BMI may not be an accurate surrogate of the obesity–mortality association (24). Third, standard BMI-derived obesity categories may not correctly represent the true distribution of obesity in Mexican Americans and using cut points from data based on ancestry-specific BMI distributions may better predict mortality (25). However, using the standard BMI-derived obesity categories allows for comparison to previous studies. Fourth, NHANES does not contain sufficient data from other Hispanic subgroups (eg, Cubans, Dominicans) to estimate their BMI–mortality associations. Understanding this association among other Hispanic subgroups would be beneficial in public health intervention planning, taking into account differences based on country of origin. Fifth, because of the way NCHS collapsed all causes of death into the top 10 leading cause-of-death categories in the 2011 public-use mortality release we could not examine other specific leading causes of death for Hispanics, specifically unintentional injuries and liver disease (15). In addition, NHANES does not ask participants if they intentionally lost weight before time at survey. Our interpretation of the results assumes that when using the maximum lifetime BMI approach, all or most observed weight loss is unintentional versus intentional. Lastly, although results indicated an increased risk of mortality among some BMI classes, caution should be used in extrapolating these findings with respect to illness.

Using maximum lifetime BMI derived from a weight history to model the BMI–mortality association in Mexican American adults indicated that, after accounting for individuals who lost weight over time, those classified as obese class II had an increased risk of all-cause mortality. Given the continued increase in the Hispanic population, working to better understand the complex associations between obesity, obesity-related chronic illness, and death is imperative to inform future efforts to promote health and reduce disease among this population.
